# Biomarkers of aging associated with past treatments in breast cancer survivors: when therapy-induced pathways turn out to be potential therapeutic targets

**DOI:** 10.1038/s41523-018-0058-6

**Published:** 2018-03-06

**Authors:** Wafaa Jabbour, Didier Wion

**Affiliations:** 1 Mansourieh Laboratory, Mansourieh, Lebanon; 2 INSERM U1205, BrainTech Lab, Université Grenoble Alpes, Domaine de la Merci, 38700 La Tronche, France

In a recent paper in *NPJ Breast Cancer*, Scuric et al.^1^ found that in breast cancer patients, chemotherapy and radiation exposure were associated with some biomarkers of aging in white blood cell (WBC) and in peripheral blood mononuclear cells (PBMCs). Elevated levels of sTNF-RII, a biomarker of pro-inflammatory activation, were also detected. These results were obtained 3–6 years after the completion of primary treatments.

It has long been known that chemotherapy and/or radiotherapy can directly enhance the risk of secondary malignancy by increasing the risk of cumulative cellular mutations.^2^ However, the long-lasting effects of chemotherapy and/or radiotherapy on WBCs and PBMCs described by Scuric et al. suggest that these effects might not exclusively result from direct DNA damage by therapy. This points to an additional mechanism: the secondary induction of a chronic subclinical state of low-grade inflammation and low-level oxidative stress. This chronic state could occur when primary DNA damage, oxidative stress, and inflammation initially induced by chemotherapy and /or radiotherapy^3^ exceed the resolving capacity of the tissue and/or organism. In this mechanistic framework, a reciprocal connection exists between low levels of oxidative stress, DNA lesions, and unresolved low-grade inflammation.^4^ With regard to DNA lesions, it should be noted that radiation-induced delayed bystander-like effects can produce DNA damage in non-irradiated cells as a consequence of communication with irradiated cells.^3,5^ Altogether, this would generate a perpetual loop that can account, at least partially, for the long-lasting induction of the biomarkers of aging observed by Scuric et al.

The key point we want to address here is that this vicious circle would be amenable to therapy. Antioxidant therapies, nonsteroidal anti-inflammatory drugs, and/or specialized pro-resolving mediators of inflammation (SPMs) may be considered. SPMs are particularly interesting. They are lipid mediators that both resolve inflammation and promote host defense.^6^ This dual role distinguishes them from immunosuppressive molecules, and therefore, SPMs represent a novel approach for treating therapy-induced tumor growth or recurrence.^7,8^ Indeed, SPMs such as resolvins have entered clinical development for inflammatory diseases.^8^ Most recently, they were also shown efficient at suppressing therapy-induced tumor growth in a preclinical model.^8^ It would be especially relevant to assess whether these drugs could dampen the biomarkers of aging associated with past radiotherapy and/or chemotherapy, and limit or delay the onset of secondary malignancies as patients age.

It may seem counterintuitive to consider that cancer therapies could contribute to the relapse of the disease they were intended to treat.^9^ However, accumulating evidence suggest that efficient treatment regimens, as they are currently delivered, can also have multiple long- and late-term pro-tumorigenic side effects,^3,8,10^ some not yet discovered. Given the continuous growth in the number of cancer survivors, this is an issue we urgently need to address.

Indeed, a long-awaited paradigm shift is occurring: therapy-induced pathways are now being considered as potential therapeutic targets.
